# Surgical Aortic Valve Replacement to Treat Prosthetic Valve Endocarditis After Valve-in-Valve Transcatheter Aortic Valve Replacement

**DOI:** 10.7759/cureus.38021

**Published:** 2023-04-23

**Authors:** Nihar Jena, Kinjal Patel, Ronak Desai, Nazia Siddiqui, Guneet Ahluwalia, Abdul R Halabi, Charles Schwartz, Sandeep Krishnan

**Affiliations:** 1 Cardiovascular Medicine, St. Joseph Mercy Oakland Hospital, Pontiac, USA; 2 Anesthesiology, Cooper University Hospital, Camden, USA; 3 Anesthesiology, Henry Ford Health System, Detroit, USA; 4 Cardiothoracic Surgery, St. Joseph Mercy Oakland Hospital, Pontiac, USA; 5 Anesthesiology, Wayne State University School of Medicine, Detroit, USA

**Keywords:** transesophageal echocardiography, surgical aortic valve replacement, transcatheter aortic valve replacement, valve-in-valve, prosthetic valve endocarditis

## Abstract

Prosthetic valve endocarditis (PVE) is an uncommon complication after heart valve replacement surgery that can result in increased morbidity and mortality. Current guidelines for management of PVE recommend antibiotic therapy followed by surgical valve replacement. The number of aortic valve replacements is expected to rise in the coming years with the expanded indications for use of transcatheter aortic valve replacement (TAVR) in patients with low, intermediate, and high surgical risk, as well as in patients with a failed aortic bioprosthetic valve. Current guidelines do not address the use of valve-in-valve (ViV) TAVR for management of PVE in patients who are at high risk for surgical intervention. The authors present a case of a patient with aortic valve PVE after surgical aortic valve replacement (SAVR); he was treated with valve-in-valve (ViV) TAVR due to the high surgical risk. The patient was discharged, but he returned to the hospital with PVE and valve dehiscence 14 months after ViV TAVR, after which he successfully underwent re-operative SAVR.

## Introduction

Prosthetic valve endocarditis (PVE) is an infrequent complication of heart valve replacement surgery. Despite significant advances in diagnosis and management, PVE is associated with increased morbidity and mortality [1]. The most common cardiac complications of PVE include heart failure from valve dehiscence, myocardial infarction from emboli, perivalvular abscess formation, intracardiac fistula, and pericarditis. Non-cardiac complications include stroke, encephalopathy, seizure, renal failure, and pulmonary embolism; these complications are usually the result of persistent bacteremia or an embolic event [2]. In 2019, the number of aortic valve replacements (transcatheter aortic valve replacements (TAVR) and surgical aortic valve replacements (SAVR)) in the United States totaled over 130,000. With the volume of aortic valve replacements expected to continue to rise in the coming years, it is likely that the incidence of PVE will also grow. The authors present the case of a patient who presented with aortic valve PVE after SAVR and was treated with valve-in-valve (ViV) TAVR due to significant surgical risk. The patient returned to the hospital with PVE and valve dehiscence 14 months after ViV TAVR and successfully underwent re-operative SAVR. 

## Case presentation

A 78-year-old male with a past medical history of chronic kidney disease stage III, hyperlipidemia, hypertension, and multiple aortic valve interventions presented to the emergency department (ED) with progressively worsening dyspnea and bilateral leg edema.

**Figure 1:**
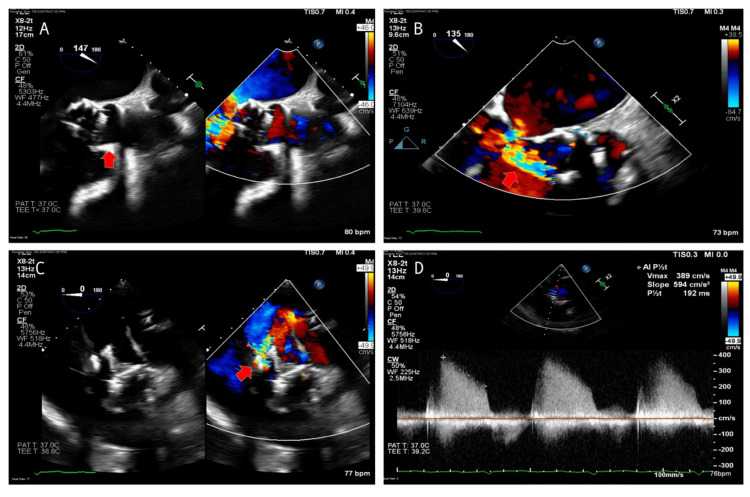
TEE of the prosthetic aortic valve structure. (A) Mid-esophageal (ME) long axis view (red arrow-anterior aortic valve annulus) showing valve structure dehisced from the anterior annulus. (B) ME long axis view zoomed (red arrow-paravalvular regurgitant flow) showing paravalvular regurgitant jet. (C) Deep transgastric (DTG) view (red arrow-paravalvular regurgitant flow) showing valvular structure dehiscence and paravalvular regurgitant jet. (D) Continuous wave Doppler measurement (pressure half-time 192 ms) consistent with severe aortic regurgitation. TEE: transesophageal echocardiogram.

The patient reported feeling short of breath with exertion and stated that his primary care physician asked him to go to the ED after a chest x-ray showed pleural effusions. On admission, the patient was afebrile with a blood pressure of 112/55 mm Hg, heart rate of 89 beats per minute, and respirations of 23 per minute. Physical examination revealed a loud grade 4/6 diastolic murmur at the second intercostal space, diffuse crackles with auscultation, and 3+ bilateral lower extremity edema. Initial laboratory results showed a hemoglobin of 9.8 g/dL, white blood cell count of 5,900 K/mcL, B-type natriuretic peptide was 901 pg/mL (normal <100 pg/mL), and high sensitivity troponin was 25 ng/L (normal <14 ng/L). A repeat chest x-ray revealed bilateral pulmonary edema and a left-sided pleural effusion. A computed tomography (CT) angiogram of the chest was negative for pulmonary embolism, and an electrocardiogram showed sinus rhythm with nonspecific ST segment abnormalities, unchanged from a prior ECG. A transesophageal echocardiogram (TEE) was performed and revealed a preserved left ventricular ejection fraction and severe paravalvular aortic regurgitation; the prosthetic valve structure was noted to be “rocking” (Figure 1, Videos 1-4).

**Video 1:** TEE DTG view showing aortic prosthetic valve structure dehiscence and paravalvular regurgitant jet. TEE: transesophageal echocardiogram, DTG: deep transgastric.

**Video 2:** TEE DTG view zoomed showing aortic prosthetic valve structure dehiscence. TEE: transesophageal echocardiogram, DTG: deep transgastric.

**Video 3:** TEE ME long-axis view showing valve structure dehiscence with a paravalvular regurgitant jet. TEE: transesophageal echocardiogram, ME: mid-esophageal.

**Video 4:** TEE ME aortic valve short-axis view showing rocking motion of the dehisced valvular structure. TEE: transesophageal echocardiogram, ME: mid-esophageal.

Further investigation into the patient’s cardiac history revealed that the patient had previously presented with severe aortic stenosis and had undergone SAVR six years prior using a 25-mm Edwards Magna Ease valve (Edwards Lifesciences, Irvine, California, USA). Five years post-SAVR, the patient developed prosthetic valve *Enterococcus faecalis* endocarditis. He was treated with antibiotics for six weeks based on culture sensitivities, and subsequent blood cultures were negative. Eight weeks after completing antibiotic treatment, he presented with severe heart failure. TEE at that time revealed severe aortic regurgitation secondary to prosthetic valve leaflet degradation without a paravalvular leak. Cardiology and cardiothoracic surgery teams evaluated the patient, and due to patient preference and prohibitive surgical risk, the decision was made to proceed with ViV TAVR after clinical recovery. The patient underwent an uncomplicated ViV TAVR with a 26-mm Edwards Sapien 3 Ultra valve (Edwards Lifesciences, Irvine, California, USA). He tolerated the procedure well and was discharged home on post-operative day (POD) three. He followed up regularly with his cardiologist and was in his usual state of health for 14 months until his clinical condition deteriorated, and he returned to the ED for his current admission.

**Figure 2:**
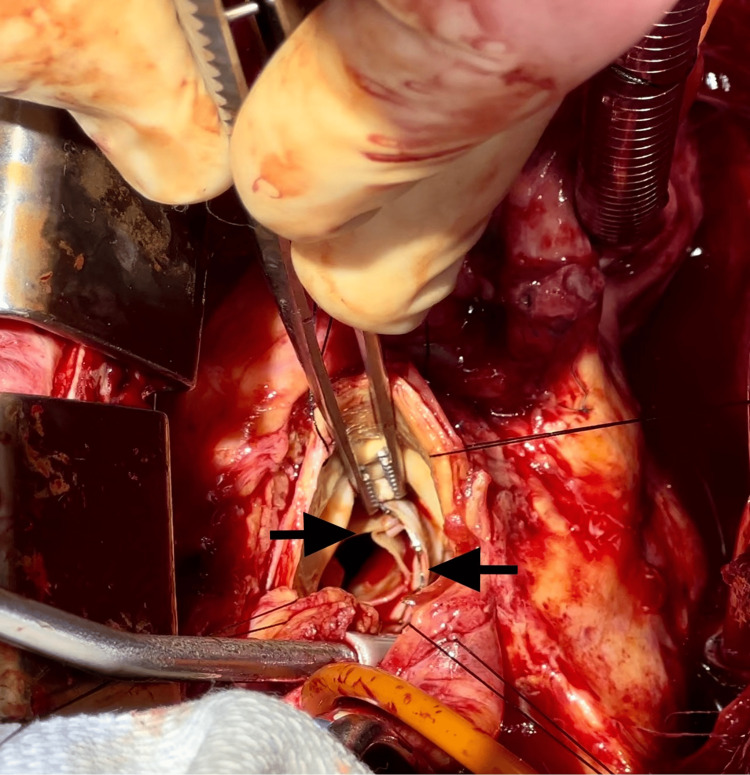
ViV TAVR leaflets with stainless-steel frame prior to explantation (black arrows). TAVR: transcatheter aortic valve replacement, ViV: valve-in-valve.

**Figure 3:**
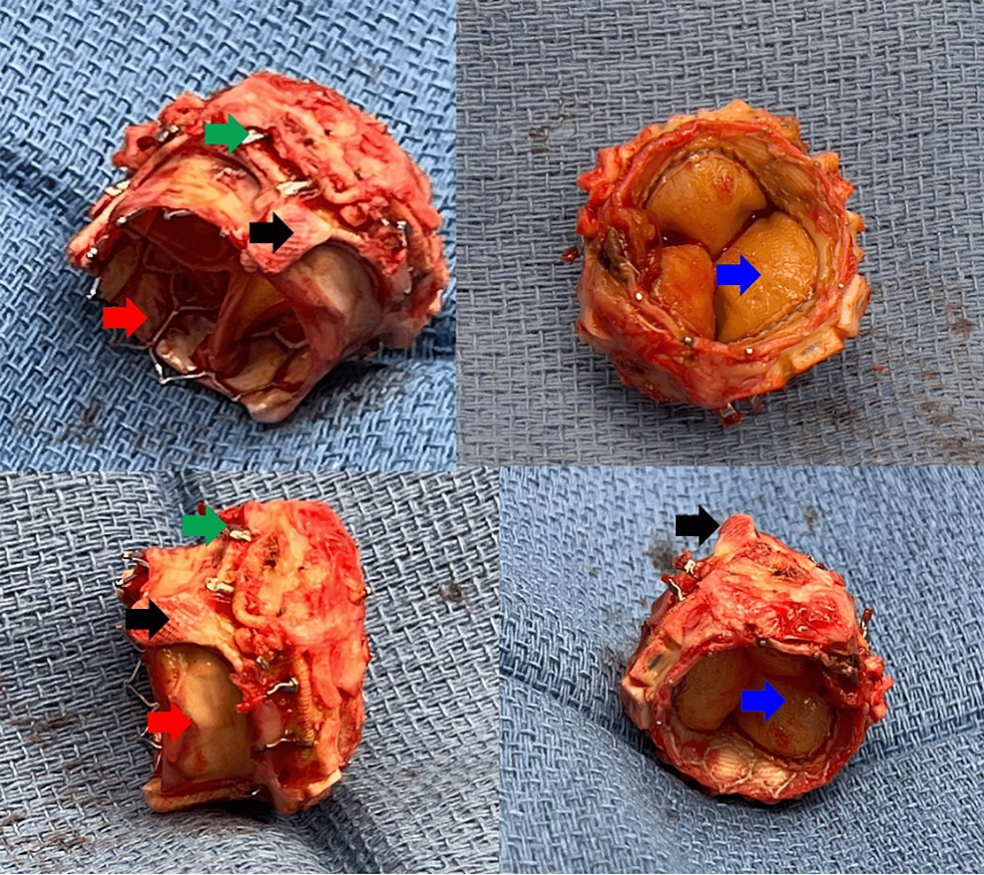
Explanted prosthetic structure. Red arrow: TAVR valve stainless steel frame, blue arrow: TAVR valve leaflets, black arrow: SAVR valve struts, green arrow: SAVR valve annulus. TAVR: transcatheter aortic valve replacement, SAVR: surgical aortic valve replacement.

On this admission, the patient was admitted to the coronary care unit for close hemodynamic monitoring; blood cultures were drawn, and broad-spectrum antibiotics were administered for presumed PVE. The patient remained hemodynamically stable, but his blood pressure revealed a widened pulse pressure. Due to the patient’s clinical presentation and TEE findings, the decision was made to pursue a re-operative SAVR. Blood cultures eventually returned positive for *Staphylococcus epidermidis*, and daptomycin and ceftriaxone were initiated with a plan to continue them for six weeks. Repeated blood cultures were negative. On the day of surgery, the patient was brought to the operating room and placed under general anesthesia. Cardiopulmonary bypass was initiated, and upon surgical exploration, the TAVR valve was noted to have intact leaflets and was firmly attached to the surgical valve (Figures 2, 3).

The anterior portion of the previously implanted surgical valve was observed to have dehisced from the aortic annulus near the right and non-coronary cusps. Excision of the valvular structure caused a small aorto-atrial (left) fistula near the left and non-coronary cusps, and this defect was repaired using a bovine pericardial patch. A 25-mm Edwards Inspiris Resilia bioprosthetic valve (Edwards Lifesciences, Irvine, California, USA) was implanted without complication (Videos 5, 6).

**Video 5:** TEE ME right ventricular inflow/outflow view, replaced the aortic valve with a mild intravalvular regurgitant jet. TEE: transesophageal echocardiogram, ME: mid-esophageal.

**Video 6:** TEE ME long-axis view showing a mild intravalvular regurgitant jet with a well-seated and functioning valve. TEE: transesophageal echocardiogram, ME: mid-esophageal.

The patient tolerated the intraoperative procedure well; however, upon return to the surgical intensive care unit (SICU), the patient became hemodynamically unstable with increased vasopressor requirements and increased chest tube output. A massive transfusion protocol was initiated, and an open sternotomy at the bedside was performed that revealed no obvious source of continued bleeding. Due to his guarded condition, the patient’s chest remained open for 36 hours, after which the patient returned to the operating room for chest closure. The patient’s condition continued to improve, and he was extubated on POD four, transferred to the cardiology floor from the SICU on POD 11, and then discharged to a subacute rehabilitation facility on POD 14. He was discharged to home on POD 35 in good condition and has consistently followed up as an outpatient.

## Discussion

Prosthetic valve endocarditis (PVE) remains a rare and life-threatening complication after valve replacement. The incidence of PVE in SAVR and TAVR is reported to be 4.81-5.28/per 1000 patients and 3.57-3.74/per 1000 patients, respectively [3,4]. Several studies have documented common risk factors for the development of PVE post-SAVR and post-TAVR; these risk factors include younger age, male sex, diabetes mellitus, severe pulmonary disease, renal insufficiency, pulmonary hypertension, prior cancer or chest radiation, liver cirrhosis, higher body mass index, and at least moderate residual aortic regurgitation post-TAVR [5]. The patient described had a history of chronic kidney disease, which may have increased his risk of PVE.

Diagnosis of PVE can be more difficult than that of native valve infective endocarditis (IE). While the preferred initial imaging modality to diagnose suspected native valve IE is transthoracic echocardiography (TTE), TEE is recommended for patients with suspicion for PVE due to superior image quality. For many years, the modified Duke criteria have been widely used to diagnose native valve IE. Increased use of prosthetic valves during SAVR and TAVR, however, has caused many to question the sensitivity and specificity of the Duke criteria in diagnosing PVE and, specifically, the limitations of echocardiography in the evaluation of PVE. Additionally, current guidelines for diagnosis are limited to PVE with a single valve. Alternative imaging techniques are now being considered for the evaluation of PVE, including cardiac CT with or without angiography, magnetic resonance imaging, fluoro-18-fluorodeoxyglucose positron emission tomography (PET)/CT, and radiolabeled white blood cell single photon emission tomography (SPECT)/CT, among others. Scientific evidence related to various imaging modalities remains limited, though, and current guidelines addressing their use vary widely across geographic regions [6].

Current principles for management of patients presenting with PVE focus on antibiotic therapy and surgical intervention. The 2020 American College of Cardiology/American Heart Association (ACC/AHA) guidelines for management of valvular disease recommend antibiotic therapy prior to surgical treatment (SAVR) for patients presenting with suspicion for PVE and further state that management with transcatheter therapy is contraindicated (11.9.3) [7]. Similarly, the American Association for Thoracic Surgery (AATS) guidelines for management of PVE also recommend an antibiotic regimen and surgical therapy (IIa). However, AATS guidelines also emphasize that “factors related to complications should be considered” in the decision-making process [8]. To date, there are no specific guidelines for management of ViV PVE. On presentation after his initial SAVR, our patient was deemed to be at high surgical risk due to his comorbidities and clinical condition by both the cardiology and cardiothoracic surgical teams. Additionally, he insisted on TAVR intervention rather than an open surgical approach. Unfortunately, a second episode of PVE with prosthetic valve structure dehiscence and the risk of prosthetic valve embolization necessitated surgical intervention.

There are a few reported cases of PVE treated with ViV TAVR. Shen et al. described the use of TAVR for management of native valve IE in an 88-year-old man with lymphocytic lymphoma and severe aortic regurgitation requiring inotrope and vasopressor support. He was started on an antibiotic regimen, and after the multi-disciplinary team and family discussions, it was determined that SAVR would not be attempted; the patient would instead undergo TAVR with palliative intent. The patient underwent TAVR without complication and was discharged on POD 13; he finished his antibiotic course and was reported to be healthy and functionally active six months later [9]. The case report highlights native valve IE successfully treated with TAVR instead of SAVR; TAVR was not performed to cure his endocarditis but was performed with the goal of weaning the patient from hemodynamic support and allowing him to return home. A Cardiothoracic Surgery Case Conference from Brigham and Women’s Hospital discussed a middle-aged male who received ViV TAVR after SAVR with root repair that was complicated by late-stage PVE. The patient presented after SAVR with fever, tachycardia, tachypnea, and hypoxia. He was deemed high-risk for SAVR and was successfully treated with TAVR ViV and empiric antibiotics; he was eventually discharged home and was clinically stable in follow-up [10]. The described case is similar to our patient in that both patients acquired PVE after their initial SAVR. Both patients were also evaluated by cardiology and cardiothoracic surgery and considered to be high-risk surgical valve replacement candidates, after which they underwent ViV TAVR. Our patient’s clinical course differed in that he had a subsequent episode of PVE that affected the prosthetic valve structure.

The frequency of TAVR ViV procedures is steadily rising due to advancements in valve delivery techniques and increasing operator experience, coupled with an aging population and the expanded use of TAVR in low- and medium-risk populations. Current ACC/AHA and AATS guidelines recommend SAVR as the definitive intervention for PVE, but re-operative SAVR can be a risky endeavor. Re-operative cardiac surgery has known risks, including injury to previously placed bypass grafts, severe bleeding, and direct injury to the heart, lungs, and great vessels; re-operative cardiac surgery for IE also carries the risk of additional surgery above and beyond isolated valve replacement including root replacement. Sà et al. recently conducted a meta-analysis on ViV TAVR versus redo SAVR for structural valve degeneration; they included 16,207 patients from 12 studies and found that ViV TAVR was associated with decreased 30-day mortality (OR: 0.52; 95% CI: 0.39-0.68; p < 0.001), lower rates of major bleeding (OR: 0.48; 95% CI: 0.28-0.80; p =0.013), and a shorter hospital stay (OR: −3.30; 95% CI: −4.52 to −2.08; p < 0.001). They did, however, find no statistical difference for one-year mortality and found that ViV TAVR was associated with increased rates of myocardial infarction and patient-prosthesis mismatch [11]. Malvindi et al. reported on the outcomes of patients with acute PVE and found that mortality after re-operation versus medical treatment was 17% versus 61%, and survival at one year for re-operation versus medical treatment was 78% versus 17% [12]. Current evidence suggests improved outcomes after surgical intervention for PVE when compared to medical treatment alone, but the future investigation should determine whether ViV TAVR could be a suitable alternative in high-risk patients.

## Conclusions

With an aging population, the volume of aortic valve interventions will continue to rise; consequently, there will likely be a concomitant increase in PVE. It would be prudent to gain an understanding of whether ViV TAVR is a viable choice for select high-risk patients with PVE. Further investigation is warranted to help establish updated guidelines for management of PVE. As in this case, a team approach to management and treatment will be important in improving outcomes.
